# Fast-track versus conventional surgery in relation to time of hospital discharge following total hip arthroplasty: a single-center prospective study

**DOI:** 10.1186/s13018-021-02640-x

**Published:** 2021-08-12

**Authors:** Raul Frankllim de Carvalho Almeida, Humberto Oliveira Serra, Liszt Palmeira de Oliveira

**Affiliations:** 1 University Hospital of Federal University of Maranhão (HU/UFMA), São Luís, MA Brazil; 2 Department of Medicine II, Federal University of Maranhão (UFMA), São Luís, MA Brazil; 3 Department of Orthopedics, School of Medical Sciences, State University of Rio de Janeiro (UERJ), Rio de Janeiro, RJ Brazil

**Keywords:** Acetabulofemoral joint, Osteoarthritis, Patient discharge, Orthopedic procedures, Enhanced recovery after surgery

## Abstract

**Background:**

Total hip arthroplasty (THA) has been used for over five decades for treating hip osteoarthritis. THA is a surgical procedure associated with prolonged hospital length of stay (LOS). The aim of this study was to analyze whether a protocol developed for fast-track THA could decrease the time taken to reach functional recovery after surgery and the hospital LOS. Blood transfusion and critical care requirements and the complication rate were evaluated as secondary endpoints.

**Methods:**

Ninety-eight patients underwent THA at the University Hospital of the Federal University of Maranhão (São Luís, Brazil). The control group included 51 patients who underwent THA through the conventional method. The fast-track surgery (FTS) group included 47 patients who underwent THA through the FTS approach. The inclusion criteria were that the subjects needed to present hip osteoarthritis and at least one clinical indication for THA, and that their risk classification was in ASA category I or II. The following factors were evaluated: age, sex, diagnosis, laterality, type of arthroplasty, blood transfusion, critical care requirement, complications, LOS, and need for re-hospitalization for any reason. For spinal anesthesia, an opioid-free protocol was used. Comparison of categorical variables between the groups was performed using the chi-square test, Shapiro-Wilk test, Student *t* test, and Poisson regression approach.

**Results:**

The FTS and control groups were similar in age and sex distribution (*p* > 0.05). The majority of the patients in the control group required both blood transfusion and use of the critical care unit, thus differing from the patients who underwent FTS (*p* < 0.001). The mean hospital LOS in the FTS group was 2.3 ± 0.8 days, compared with 6.4 ± 1.5 days in the control group (p < 0.001).

**Conclusion:**

Use of FTS was associated with decreased LOS, compared with conventional THA.

**Trial registration:**

https://www.researchsquare.com/article/rs-369025/v1.

## Background

Total hip arthroplasty (THA) has been used for over five decades for treating hip osteoarthritis, with the aims of reducing pain levels and ameliorating articular function and quality of life [1]. Furthermore, it has become widely utilized due to population aging [2]. Nevertheless, THA is generally considered to be a traumatic surgical procedure and it has been associated with intraoperative blood loss, intense postoperative pain, requirement for intensive care unit (ICU) use, and prolonged immobilization. This complex scenario may therefore delay patients’ functional rehabilitation and prolong their hospital length of stay (LOS) [3].

Novel evidence-based protocols targeting surgical patients’ care emerged in the early 1990s, with the aim of accelerating postoperative recovery. These were put forward by Professor Henrik Kehlet for use within colorectal surgery. This approach is named fast-track surgery (FTS) or enhanced recovery after surgery (ERAS) [4]. FTS is an evidence-based multimodal approach that can reduce convalescence time and improve clinical outcomes, including reductions in both morbidity and mortality, thereby significantly reducing the time taken to reach hospital discharge and optimizing patient satisfaction. The main strategy consists of five steps: careful patient selection, improving preoperative care, minimizing perioperative stress, decreasing postoperative discomfort and improving postoperative recovery. This has the potential to lead to lower mortality and morbidity, along with optimizing patient satisfaction [5–12].

On the other hand, use of FTS has also been linked to increased rates of complications and readmissions [13]. Nonetheless, several studies have shown that a reduction in hospital LOS does not compromise patient safety, compared with conventional treatment [5, 11, 14]. Early postoperative mobilization associated with prophylaxis for short-term deep venous thrombosis has also been shown to decrease the incidence of thromboembolic events [9, 15–17].

The increasing worldwide demand for arthroplasties is mainly due to the marked augmentation of the population’s longevity over recent decades, with subsequent elevation of public healthcare costs [3, 18, 19]. In this context, the aim of the present study was to analyze whether a protocol developed for fast-track THA could decrease the time taken to reach functional recovery after surgery and the hospital LOS.

## Methods

### Participants, place, and period of the study

This study was approved by our institution’s ethics committee under the number 1.829.945. The inclusion criteria were, for both conventional and FTS group, that the subjects needed to present hip osteoarthritis and at least one clinical indication for THA, and that their risk classification was in American Society of Anesthesiologists (ASA) score I or II. Patients who met the inclusion criteria and agreed to participate in the study received both oral and written information about the study purposes, signed an informed consent statement, and were then included in the study. Patients with decompensated comorbidities (ASA III or IV) or neurological disease, and those who underwent THA due to hip fracture, infection, or revision of previous THA were not included in the study. We also excluded patients for whom it was not possible to perform THA using spinal anesthesia.

A total of 98 patients underwent THA at the University Hospital of the Federal University of Maranhão (São Luís, Brazil). These patients were allocated into two groups. The control group included patients who underwent THA through the conventional method, and who were operated between January 2012 and November 2016. The FTS group included patients who underwent THA through the FTS approach, in the period from December 2016 to January 2018.

A single surgeon performed the operations on all participants. Surgery was performed on a standard operating table without special attachments, through a posterolateral approach under spinal anesthesia. Prophylaxis against infection was implemented using cefazolin (2 g), starting 30 min before induction of anesthesia and continuing for 48 h. Prophylaxis against venous thromboembolism was identical in both groups, and consisted of use of low molecular weight heparin (enoxaparin, 40 mg/day) during hospitalization and maintained through use of oral anticoagulant (rivaroxaban, 10 mg/day) for four weeks after hospital discharge. The outpatient follow-up included a 15-day return for suture removal and x-ray control, and subsequent return visits after 1, 3, 6, and 12 months.

The following factors were evaluated: age, sex, ASA score, hypertension, diabetes, obesity, diagnosis, laterality, type of arthroplasty, complications, blood supply, ICU postoperative support, LOS, and need for re-hospitalization for any reason. The complications investigated included deep or superficial infection, thromboembolic events, mortality, pneumonia, urinary tract infections, intraoperative fracture, renal failure, or any other medical conditions that could result in prolonged hospital stay.

### Conventional protocol

A total of 51 patients underwent conventional THA. In the protocol used, the patients and their families did not receive any detailed written information about the surgery; a multidisciplinary approach was not encouraged; opioids were routinely used for induction of anesthesia, spinal anesthesia, and postoperative pain management; a bladder catheter was routinely utilized; use of ICU support was mandatory; and functional rehabilitation started from the first or second postoperative day. There was no clearly defined hospital discharge goal.

### FTS protocol

A total of 47 patients underwent THA through the FTS approach. Detailed information was provided to the participants and their family members regarding the surgical procedure, potential complications, rehabilitation, and the intent to reach hospital discharge within three days after surgery [20]. A multidisciplinary team, including physicians, social service, nursing team, a physiotherapist and a dentist provided support for the patients’ care.

For spinal anesthesia, an opioid-free protocol using bupivacaine (0.5%) and clonidine (15 μg) was used. Delayed bladder catheterization was not performed. Prophylaxis against blood loss consisted of administering TA (15–20 mg/kg) before induction of anesthesia. A combination of ropivacaine (2 mg/mL), ketoprofen (100 mg), and methylprednisolone (40 mg) was infiltrated as a peri-articular injection (PAI).

After the surgical procedure, the patients then underwent a rehabilitation stage comprising mobilization in bed, within the first 6 h after surgery. They were encouraged to sit up for about 30 min, and then underwent orthostatic and gait training according to their pain tolerance. These rehabilitation procedures were dependent on absence of nausea, vomiting, malaise, dizziness, or hypotension.

The criteria for hospital discharge took into account the patients’ clinical stability, spontaneous mobilization in bed, acceptance of oral diet, pain control with oral medication, and desire to go home.

The differences between the conventional and FTS protocols are summarized in Table 1.

**Table 1:** Differences between the conventional and fast-track surgery protocols

Intervention	Conventional	FTS
Patient education	✕	✓
Teamwork concept	✕	✓
Dentist support	✕	✓
Tranexamic acid	✕	✓
Spinal anesthesia with opioid	✓	✕
Bladder catheter	✓	✕
Peri-articular injection	✕	✓
Intensive care unit	✓	✕
Rehabilitation in the first 6 h	✕	✓
Hospital discharge after fewer than 3 nights	✕	✓

### Statistical analysis

Comparison of categorical variables between the groups was performed using the chi-square test. The normality of numerical variables was tested using the Shapiro-Wilk test, and means ± standard deviations were compared using the Student *t* test. For multivariate analysis, a generalized linear model was developed, in which the number of days from surgery until hospital discharge was taken to be the dependent variable. Other variables were used as independent variables in the model, and all of them were forced into the equation. A Poisson regression approach was then used to obtain incidence rate ratios (IRRs) and confidence intervals (CIs). The data were analyzed in the SPSS Statistics for Windows software, version 24.0 (IBM Corp, Armonk, NY, USA). The significance level was taken to be 5% for all analyses.

## Results

A total of 98 individuals were included in the current study, among whom 51 underwent conventional THA and 47 underwent fast-track THA. A comparison of sociodemographic and clinical data is presented in Table 2.

**Table 2:** Sociodemographic and clinical characteristics compared between patients who underwent FTS and those who underwent conventional THA.

Variable		Conventional		FTS		*p* value
		*n*	%	*n*	%	
Sex	Male	25	49	25	53.1	0.68
	Female	26	51	22	46.8	
Age range	≤ 49 years	13	25.4	15	32	0.70
	50–59 years	17	33.3	16	34	
	≥ 60 years	21	41.1	16	34	
ASA score	Type I	*27*	*52.9*	*24*	*51*	0.85
	Type 2	*24*	*47.1*	23	48.9	
Hypertension		*23*	45	21	44.6	0.96
Diabetes type 2		*12*	23.5	13	27.6	0.63
Obesity		*25*	49	23	48.9	0.99
Side operated	Right	24	47.1	17	36.1	< 0.001
	Left	27	52.9	17	36.1	
	Both	-	-	13	27.6	
Cementation	Yes	27	52.9	24	51.1	0.44
	No	16	31.3	19	40.4	
	Hybrid	8	15.6	4	8.5	
Implant	Brazilian brand	48	94.1	44	93.6	0.91
	Foreign brand	3	5.9	3	6.3	
Blood transfusion	Yes	38	74.5	2	4.2	< 0.001
	No	13	25.4	45	95.7	
Intensive care unit	Yes	37	72.5	1	2.1	< 0.001
	No	14	27.4	46	97.8	
Length of stay	Up to 6 days	22	43.1	47	100	< 0.001
	7 or more days	29	56.8	-	-	

Significance level was taken to be *p* < 0.05

The FTS and control groups were similar in age distribution (*p* > 0.05), and most patients were older than 50 years. Sex distribution, ASA score, hypertension, diabetes type 2, and obesity did not differ between the two groups (Table 2). Side involvement varied between the two groups, and bilateral surgery was only performed in the FTS group (*p* < 0.05). The cementation and implant characteristics were similar between the two groups (*p* > 0.05). The majority of the patients in the control group required both blood transfusion and use of the ICU, thus differing from the patients who underwent FTS (*p* < 0.001). No patient in the FTS group stayed in hospital for more than 6 days, whereas for 56.8% of the controls, the time that elapsed until hospital discharge was seven or more days (*p* < 0.001) (Table 3). The mean hospital LOS in the FTS group was 2.3 ± 0.8 days, compared with 6.4 ± 1.5 days in the control group (*p* < 0.001).

**Table 3:** Results from the chi-square and Mann-Whitney tests.

Variables	Group		***P*** value
	Conventional (***N*** = 51)	Fast track (***N*** = 47)	
**Blood transfusion**			
Yes	38 (74.5)	2 (4.2)	0.000
No	13 (25.4)	47 (95.7)	
**Intensive care unit**			
Yes	37 (72.5)	1 (2.1)	0.000
No	14 (27.4)	46 (97.8)	
**Complications**			
No	49 (96.0)	42 (89.6)	0.124
Yes	2 (3.9)	5 (10.6)	
**Length of stay (nights)**	6.4 ± 1.5	2.3 ± 0.8	< 0.001

Significance level was taken to be *p* < 0.05

Neither in the FTS group nor in the control group were there any cases of thromboembolic events, infections, readmissions, or deaths. Five cases of surgical complications were observed in the FTS group, including one of traumatic dislocation, one of sciatic nerve partial neuropraxia and three of periprosthetic fracture. Among the conventional group, we also noticed two cases of partial sciatic nerve neuropraxia. The occurrence of such complications in both groups took place after surgery process and was not related to preexisting comorbidities.

## Discussion

In the present study, we evaluated whether FTS could reduce the hospital LOS, in comparison with conventional THA. It has been estimated that the number of THA operations performed will double within the next decade [3, 18], and the economic burden associated with this increasing demand is high. In this context, one potential way to reduce hospitalization costs is to minimize the LOS. Indeed, data from the mid-1990s showed that the LOS could be reduced (from 8 to 3.8 days) without affecting patient outcomes [21]_._

Recent advances in preoperative education, blood conservation, standardized anesthetic protocols, postoperative care with pain management, and early mobilization have led to popularization of FTS for THA [5–7]. A FTS protocol was introduced in our institution based on the best evidence for clinical practice, with the aims of increasing the safety of surgery, reducing and managing blood loss, controlling postoperative pain without adverse effects from opioids, and providing optimized rehabilitation with increased patient satisfaction and shorter hospital stay. In the present prospective single-center study, it was observed that patients who underwent FTS stayed in hospital for a significantly shorter period of time.

Additionally, the implementation of this FTS protocol has included both preoperative education for patients and their family members and the development of a multidisciplinary team that had previously not yet become integrated. The main outcome that was expected from the implementation of this protocol was a reduction in the hospital LOS, along with a decreased need for ICU support. After the introduction of the FTS protocol, the number of THA procedures performed was significantly augmented without an increase in the occurrence of complications.

One prerequisite for promoting effective rehabilitation and optimizing mobilization in bed is to achieve adequate pain control with few adverse effects. This was achieved in the present study through using an opioid-free protocol consisting of a combination of bupivacaine and clonidine, for spinal anesthesia. Clonidine is a 2-adrenergic agonist that has analgesic properties and boosts the effects of local anesthetics when administered via the epidural route, with few postoperative adverse effects [22, 23]. This intervention was previously found to enable elimination of postoperative delayed bladder catheter use [24].

In our study, a total of four cases of urinary retention occurred in the FTS group and required use of postoperative relief. In the control group, which underwent spinal anesthesia using morphine, all the patients received a delayed bladder catheter immediately after anesthesia. Other benefits of the opioid-free protocol were absence of or decreased adverse effects such as pruritus, constipation, malaise, nausea, and postoperative vomiting.

Relief of acute pain after THA is a major therapeutic challenge, given that postoperative pain hinders early mobilization and rehabilitation, which subsequently leads to longer periods of resting in bed and prolonged hospital LOS [10, 11]. Administration of cocktails containing local anesthetics, anti-inflammatory agents, and corticosteroids, as a PAI around the surgical site, can be implemented after surgery, to promote pain control without increasing the risk of surgical site infection [10, 12, 25, 26]. In the FTS group, the PAI technique was implemented at the end of the operation, and this led to the relief of acute postoperative pain, early mobilization, and decreased hospital LOS.

Postoperative anemia is a very common clinical condition observed in THA, which requires blood transfusion in 33 to 74% of the cases [27]. The costs and the risk of infection associated with blood transfusion, along with difficulty in obtaining blood products, have given rise to increased interest in blood conservation strategies [27, 28].

In the present study, in order to reduce the risks relating to blood transfusion in the postoperative period following FTS, the patients in this group were screened for transfusion risk factors and then treated when serum hemoglobin was < 12 g/dL [27]. Closed suction drains were not used in either group, given their low benefit and the increased risk of postoperative transfusion [29].

Another very useful strategy that was adopted in order to obtain good hemostasis during surgery was intravenous administration of TA in a single dose of 15–20 mg/kg, before induction of anesthesia [29]. TA is a lysine analog that inhibits activation of plasmin, thereby inhibiting tissue fibrinolysis and consequent clot stabilization [30].

This measure drastically reduced the need for blood transfusion in the FTS group. Blood transfusion was only required in two patients. One of these patients had sickle cell anemia and the other had rheumatoid arthritis. Both of them showed acute signs of anemia, confirmed by laboratory tests, which therefore required two units of erythrocyte concentrate each. They underwent rehabilitation on the second postoperative day and remained hospitalized for four nights after surgery. It is important to note, in comparison, that as many as 74.5% of the patients in the control group required blood transfusion. Blood transfusion is considered to be an important predictor in relation to hospital discharge [21, 27]. Prior to the introduction of FTS at the HU-UFMA, we did not have an established protocol for the prevention and management of blood loss. Patients were operated with hemoglobin levels below 12/dl and we did not use TA. Compared with the current FTS protocol, the use of conventional THA was associated with greater need for supplies of blood products.

Only one patient in the FTS group required the use of the ICU in the postoperative period. However, this was a preoperative indication based on the individual’s advanced age. The majority (72.5%) of the patients in the control group required critical care support. Neither in the FTS group nor in the control group occurred any cases of thromboembolic events, infections, heart attack, readmissions, or deaths.

Regarding the surgical complications observed in the FTS group, one case of sciatic nerve paresthesia and one case of traumatic dislocation of THA were observed. These patients underwent appropriate treatment and stayed in hospital for 5 days. Two cases of periprosthetic fracture of type Vancouver B1 and one case of type Vancouver Al were radiographically identified in the immediate postoperative period. These patients then underwent conservative treatment with a 6-week load restriction. Another case of fracture was identified at the level of the minor trochanter. The time until hospital discharge in these cases ranged from three to five days, and all of these patients evolved well, with fracture healing and no requirement for reoperation. In the conventional group, two cases of partial sciatic nerve paresthesia occurred. We emphasize that such events in both groups were caused by surgical procedures, therefore, there is no direct connection between FTS program implementation and these complications.

Another factor that needs to be observed is whether there is any family support [31]. In the FTS group, we had two patients who stayed in hospital for four nights due to social need. These were patients with social problems, who were eligible for discharge after the second night after their surgery, but they lived in other cities and could not be discharged because they had no support for them to leave the hospital. This increased their hospital LOS to four nights. We called these cases “social internment.”

The time needed to reach hospital discharge in THA cases is a subject of great debate, and there is no consensus on the ideal criteria that should be met for safe hospital discharge. Normal vital signs, adequate pain control, and safe mobilization are usually considered to be requirements [32]. However, blood loss should also be taken into account, and patients with intraoperative blood loss < 500 mL or with hemoglobin > 9.7 g/dL may thus be considered eligible for hospital discharge [32]. In the FTS protocol used, we adopted an evidence-based criterion for hospital discharge [21, 32–34].

The hospital LOS in our study was notably lower in the FTS group (mean 2.3 nights; range, 2 to 5 nights) than in the control group (mean 6.4 nights; range, 3 to 9 nights. The FTS group also had lower requirements for blood transfusion and ICU support during the postoperative period.

Overall, in addition to adequate pain control and mobilization in bed within the first postoperative hours, an absence of clinical changes (e.g., malaise, nausea, or vomiting), stability of vital signs, a desire to go home, and the existence of family support are relevant for achieving safe hospital discharge [31, 35, 36].

It needs to be mentioned that this study had several limitations, including the small sample size, the evaluation at a single center, and the fact that interventions were not performed in a parallel fashion in the two groups. Prospective multicenter cohort studies in this regard are thus warranted.

## Conclusion

The use of FTS was associated with reduced hospital LOS, compared with conventional THA. FTS consisted of multimodal and multidisciplinary standardized care in which the aims were to achieve pain control, early mobility, and early discharge.

## Data Availability

The datasets used and/or analyzed during the current study are available from the corresponding author on reasonable request.

## Abbreviations

THA: Total hip arthroplasty
LOS: Length of stay
FTS: Fast-track surgery
ERAS: Enhanced recovery after surgery
ICU: Intensive care unit
ASA: American Society of Anesthesiologists
TA: Tranexamic acid
IRR: Incidence rate ratio
CI: Confidence interval
PAI: Peri-articular injection
